# Comparative Study of *Mycobacterium bovis* and *Mycobacterium avium* subsp. *paratuberculosis* In Vitro Infection in Bovine Bone Marrow Derived Macrophages: Preliminary Results

**DOI:** 10.3390/microorganisms12020407

**Published:** 2024-02-17

**Authors:** Benedetta Amato, Dorotea Ippolito, Maria Vitale, Rosa Alduina, Paola Galluzzo, Elisabetta Gerace, Flavia Pruiti Ciarello, Michele Fiasconaro, Vincenza Cannella, Vincenzo Di Marco Lo Presti

**Affiliations:** 1 Bristol Veterinary School Langford Campus, University of Bristol, Bristol BS40 5DU, UK; er21862@bristol.ac.uk; 2 Unit of Emerging Zoonoses, Department of Food Safety, Nutrition and Veterinary Public Health, Istituto Superiore di Sanità, Viale Regina Elena, 299, 00161 Rome, Italy; 3 Istituto Zooprofilattico Sperimentale della Sicilia, via S. Andrea 96, 98051 Barcellona Pozzo di Gotto, Italy; maria.vitale@izssicilia.it (M.V.); paola.galluzzo@izssicilia.it (P.G.); geraceelisabetta74@gmail.com (E.G.); pruitiflavia@outlook.it (F.P.C.); michele.fiasconaro@izssicilia.it (M.F.); vincenza.cannella@izssicilia.it (V.C.); vincenzo.dimarco@izssicilia.it (V.D.M.L.P.); 4 Dipartimento di Scienze e Tecnologie Biologiche Chimiche e Farmaceutiche, Università degli Studi di Palermo, Viale delle Scienze, 90128 Palermo, Italy; valeria.alduina@unipa.it

**Keywords:** macrophages, IL-1β, IL-6, in vitro infection, *Mycobacterium bovis*, *Mycobacterium paratuberculosis*

## Abstract

Bovine tuberculosis and paratuberculosis are endemic in many areas worldwide. This work aims to study cytokines production and gene expression profiles of bovine macrophages infected with *Mycobacterium bovis* and *Mycobacterium paratuberculosis* subsp. *avium* (MAP) strains to identify potential diagnostic biomarkers. Bovine bone marrow stem cells were differentiated into macrophages and subsequently infected in vitro with different spoligotypes of *M. bovis* and MAP field strains (as single infections and coinfections), using different multiplicity of infection. Supernatant and cell pellets were collected 24 h, 48 h, and one week post-infection. Preliminarily, gene expression on cell pellets of IL-1β, IL-2, INFγ, IL-6, IL-10, IL-12, and TNFα was assessed by qRT-PCR one week p.i. Subsequently, IL-1β and IL-6 were measured by ELISA and qRT-PCR to investigated their production retrospectively 24 h and 48 h p.i. A variability in macrophages response related to the concentration of mycobacteria, the coinfection with MAP, and *M. bovis* spoligotypes was identified. An early and constant IL-6 increase was observed in the *M. bovis* infection. A lower increase in IL-1β was also detected at the highest concentration of the two *M. bovis* spoligotypes one week post-infection. IL-6 and IL-1 β production was reduced and differently expressed in the MAP infection. IL-6 appeared to be the earliest cytokines produced by bovine macrophages infected with *M. bovis*.

## 1. Introduction

The genus Mycobacteria is composed by several phylogenetically related species, which can be grouped in two main complexes: the *Mycobacterium tuberculosis* complex (MTBC), that includes *Mycobacterium bovis*, *Mycobacterium caprae* and *Mycobacterium tuberculosis* among others [1], and the Mycobacteria Other Than Tuberculosis (MOTT) [2], generally recognised as environmental mycobacteria. Within the last group, avian mycobacteria belonging to the *Mycobacterium avium* complex (MAC), includes some relevant animal pathogens, such as *Mycobacterium avium* subsp. *paratuberculosis* (MAP) [3], which is responsible for the most important chronic, debilitating, contagious infection of the intestinal tract in ruminants known as Johne’s disease or paratuberculosis [4]. The disease is generally asymptomatic, but, in the advanced stages of infection, diarrhoea and cachexia may be present [5].

*M. bovis* is the causative agent of bovine tuberculosis also known as animal tuberculosis (TB) due to its capacity to infect a broad range of animal species along with humans. In Sicily, the molecular analysis demonstrated high genotypic variability in *M. bovis* field strains. In particular, spoligotyping of isolates e has shown that SB0120 and SB0841 are both the most prevalent in livestock and wildlife, whereas others, such asSB1564 has been isolated only from cattle at very low prevalence levels [6,7,8,9].

Both mycobacteria target macrophages and are able to survive and reproduce inside the phagosomes, due to the development of several escape mechanisms such as control of intracellular trafficking and cell death pathways including resistance to oxidative killing systems, phagosome-lysosome fusion inhibition, [10,11]. These features can potentially elicit a long-term chronic, inflammatory immune response that may be involved in the development of Chron’s disease in humans [12,13] and other immune-mediated diseases, such as multiple sclerosis [14].

Official intra-vitam diagnostic tests for TB include Single Intradermal Comparative Cervical Tuberculin test (SICCT) known also as TB skin test (TST) and Interferon-γ release assay (IGRA), whereas Johne’s disease is usually diagnosed through serum antibody detection by enzyme-linked immune-assay (ELISA) and faecal culture. Several factors have been identified as responsible for the suboptimal sensitivity and specificity of those tests, especially in areas with high bovine tuberculosis prevalence [15]. Immunisation with Bacille Calmette–Guérin (BCG) strain has been shown to produce variable levels of protection against *M. bovis* [16]. Furthermore, vaccinated animals may have a positive response to the TST raising the need to develop a test that is able to differentiate infected animals from vaccinated animals (DIVA test) [17]. Furthermore, early or subclinical stages of the disease or the presence of a concurrent MAP infection [18,19] may provide false-negative results to the skin test or to IGRA, thus contributing to the failure in controlling *M. bovis* spread [20]. Therefore, the development of a diagnostic test that can detect bovine tuberculosis in the early stages of the disease and discriminate between the *M. bovis* infection and MAP infection in live animals is highly recommended.

Proteomics and transcriptomics have shown that different cellular products are implicated in mycobacterial infection, some of which have been proposed as biomarkers [21,22,23]. In particular, cytokines such as Interleukin (IL) 6 has been detected during TB infection with rising levels [24,25], while others such as IL-1β showed a decreasing trend [26]. The chronic inflammatory pattern observed during the MAP infection seems to be a consequence of a Th1/Th2 immune response overlap, with a consistent increase in Th1 related molecules. However, infected cows also showed an increase in IL-10 production that is likely playing a protection role from prolonged and chronic inflammatory stimulation [27,28].

The purpose of this work was to analyse the cytokine production and gene expression related to the interaction between in vitro differentiated bovine macrophages and *M. bovis* and MAP field strains at different concentrations to evaluate possible differences in macrophage response related to the microbial load, as well as the cytokine production and gene expression during coinfection, to identify possible diagnostic biomarkers.

## 2. Materials and Methods

### 2.1. Identification of TB and Paratuberculosis Officially Free Farms and Selection of the Animals for the Experiment

Officially Tuberculosis-free (OTF) and paratuberculosis-free herds were identified within an OTF area and screened for TB and Paratuberculosis using intra-vitam assays as for the national eradication programme guidelines. TST was performed according to the Chapter 3.1.13 of 12th edition of the WOAH Terrestrial Manual, whereas IGRA for TB was performed according to the international Standard operating procedure (SOP) released from the European Union Reference Laboratory for Bovine Tuberculosis (Visavet Health Surveillance centre, Universidad Complutense de Madrid, Spain), using the commercial kit Bovigam, (ThermoFisher Scientific, Waltham, MA USA) for IGRA. The ELISA test for serum anti-MAP antibody detection (ID-screen^®^ Paratuberculosis Indirect, ID-VET, Grabels, France) was used to test for MAP exposition and was performed according to the manufacturer’s instructions. Finally, faecal culture for MAP isolation was performed according to SOP accredited as reported by the Accredia quality system in force at the Paratuberculosis Reference Centre of the Experimental Zooprophylactic Institute of Lombardy and Emilia Romagna. Three TB and paratuberculosis negative privately owned cows aged between 18 and 24 months, in good health and nutrition state (body condition score-BCS between 3 and 4 out of 5), not pregnant and not lactating were chosen within the selected negative herd. Farmers were asked to sign an informed consent to enrol the animals in the study.

### 2.2. Bone Marrow Collection and Macrophagic Culture

Bone marrow specimens (10 mL) were collected from the iliac crest of freshly slaughtered animals. Specimens were then poured in EDTA tubes and transferred at 4 °C in isothermal box to the laboratory. Samples were processed within 4–6 h post-collection. A pure macrophage culture was obtained following the protocol published by Trouplin et al., 2013 [29]. Pluribeads (Pluriselect) kit and flow cytometry were used to confirm the successful and correct isolation of a pure macrophage cell culture. The cell marker used was CD14 and the purity yielded was 94%. Macrophage cultures were finally seeded in 24 well flat bottom plates and monitored daily.

### 2.3. In-Vitro Infection with Field Strains of M. bovis and MAP

Once obtained the pure macrophage cell culture, three spoligotypes of *M. bovis* (SB0120, SB1564 and SB0841) and type C strain of MAP were used for the in vitro-infection alone or in combination. The strains were obtained from the routine diagnostic activity at the Zooprophylactic Institute of Sicily (Barcellona P.G., Italy) and the National Reference Centre for Paratuberculosis (Piacenza, Italy). The viability of mycobacteria was assessed prior to the experimental infection using an immunofluorescence staining (LIVE/DEAD^TM^, *Bac*Light, ThermoFisher Scientific). The colonies of *M. bovis* SB0120, SB1564, and SB0841 were separately spotted with a sterile loop and diluted in 2 mL of physiological solution. The bacterial clumps were disrupted by adding 2–3 mL of glass beads to the cell suspension and by vortexing twice for 60 s. One mL of supernatant was then collected into a sterile Eppendorf. Turbidity was adjusted to 1 (approximately 3 × 10^8^ bacteria/mL), 0.5 (approximately 1.5 × 10^8^ bacteria/mL) and 0.1 (approximately 0.3 × 10^8^ bacteria/mL) according to the McFarland scale, in order to obtain three scalar dilutions stated as maximum with MOI 1, intermediate with MOI 0.5 and minimum bacterial concentration with MOI 0.1. *M. bovis* strain (SB0120) and MAP were then mixed at the same three different concentrations in order to pursue coinfection. Infection was performed on 24 well flat bottom plates, adding 10 μL of bacterial suspension three times to the macrophage cultures obtaining biological triplicates for each concentration and mycobacterium as single or coinfection. A corresponding negative uninfected control has been also included for each challenge and bacterial concentration. Incubation of infected macrophages cultures was performed at 37 °C and 5% CO_2_ for two hours. Thereafter the cell cultures were washed twice with sterile Hank’s balanced salt solution and incubated at 37 °C and 5% CO_2_ using as medium DMEM/F12 enriched with 10% foetal bovine serum (FBS). At respectively 24, 48 h and 7 days post-infection the supernatant and the cellular pellet were separately collected and stored for the ELISA test (*M. bovis* SB0120 and *M. bovis* SB1564) and qRT-PCR (*M. bovis* SB0841, *M. bovis* SB1564).

Phagocytosis was evaluated by daily microscopic observation after Ziehl–Neelsen staining, according to standard operating procedures in use in the lab, providing thus additional information on bacterial load and eventual multiplication in the supernatant.

### 2.4. qRT-PCR

Pure mRNA was obtained from cell culture infected with the higher MOIand eluted in a total volume of 80 µL using the TurboCapture 96 mRNA Kit (Qiagen, West Sussex, UK), according to the manufacturer’s instructions. For qRT-PCR, a two-step protocol was used, as previously described [30]. Reverse Transcription of mRNA was carried out in a final volume of 20 µL, using the QuantiNova Reverse Transcription kit (Qiagen, West Sussex, UK), according to the manufacturer’s instructions. An initial step at 25 °C for 3 min was followed by a reverse transcription step at 45 °C for 10 min. The resulting cDNA was stored at −20 °C prior to further analysis by qRT-PCR. Gene-specific primers were designed using Primer3 software (version 4.1.0), available online, to amplify fragments of *Bos taurus* interleukin genes (IL-1β, IL-2, IFNγ, TNFα, GAPDH) or retrieved from bibliography (IL-6, IL10, IL12 p40) (Table 1). Two microliters of each cDNA sample were used in the q-PCR reaction with SYBR^®^ Green PCR Master Mix (Applied Biosystems, Waltham, MA, USA) according to the manufacturer’s instructions. Each 20 μL reaction contained 10 pmol of forward and reverse primers. The q-PCR was performed under the following conditions: 2 min at 50 °C and 10 min at 95 °C, followed by 40 cycles of 15 s at 95 °C and 1 min at 60 °C. Eventually, the melting temperature and the specificity of the PCR products were determined by running a dissociation reaction using the conditions previously described [31]. Sterile distilled water was used as a negative control in all the q-PCR assays, and each experiment was performed in triplicate. The threshold cycle values (CT) were determined with a baseline set automatically. Results were analysed using the comparative critical threshold method (ΔΔCT) in which the amount of target DNA is adjusted to an internal reference. The GAPDH gene was used as an internal reference to normalize the results [32]. In each run, four dilutions of cDNA were analysed to determine the PCR efficiency and negative controls were included.

### 2.5. Cytokines Measurement

The following cytokines were selected and measured using commercial ELISA kits (Cusabio, Houston, TX, USA) according to the manufacturer’s instructions: IL-1β, IL-6. The supernatants obtained from the macrophagic cultures, infected with the three different MOI, were processed in triplicates. The colorimetric reaction was measured by spectrophotometry as optical density (OD) at the wavelength of 450 nm, with the correction wavelength set at 600–630 nm. The standard curve for cytokines measurement was set into a range from 1000 pg/mL to 0 pg/mL with serial 4-fold dilutions and calculated as stated in the manufacturer’ s instructions.

### 2.6. Statistical Analysis

The results obtained were expressed as means of three independent experiments carried out in triplicate.

## 3. Results

### 3.1. qRT-PCR

Cytokine gene expression analysis was performed on non-infected cells and on cells infected with *M. bovis* SB0841, *M. bovis* SB1564, MAP and both bacteria at maximum concentration after one week.

**Figure 1:**
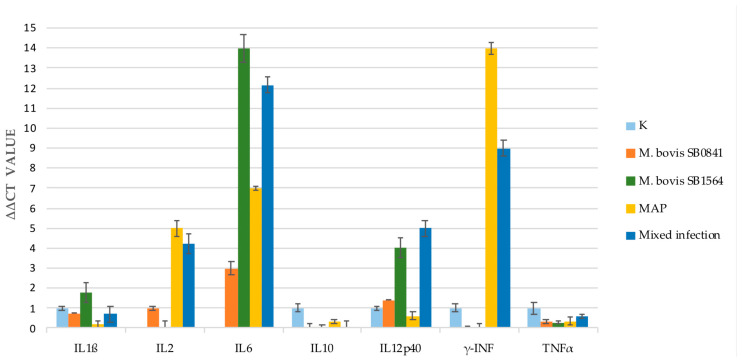
Cytokine gene expression analysis performed by qRT-PCR on non-infected cells (K) and cells infected with *M. bovis* SB0841, *M. bovis* SB1564, MAP and both bacteria (SB0841 and MAP) at maximum concentration one-week post-infection.

As shown in Figure 1 after 1 week a remarkable increase in IL-6 was observed in all the infections, with higher values for *M. bovis* SB1564 and *M. bovis*/MAP mixed infection. Conversely, *M. bovis* SB0841 elicited the lowest expression of IL-6. A slight increase in IL-1β was observed only with *M. bovis* SB1564 at one week post-infection, however gene expression was evident also in non-infected samples and other infections.

IL-2 production was not detected for *M. bovis* SB1564 after one week of infection in contrast to a slight increase for *M. bovis* SB0841 and an increase in MAP and mixed infection.

As for IFNγ, no gene expression was apparent after challenge with both *M. bovis* strains. Interestingly, the highest response was elicited by MAP infection and mixed infection, the latter with a milder gene expression.

The qRT-PCR tests of the Tumour Necrosis Factor (TNF) α and IL-10 showed a lower to absent gene expression in all the four types of infection.

Gene expression of IL-12p40 was increased in our study following infection with *M. bovis* SB1564, *M. bovis* SB0841 and mixed infection whereas low levels were detected when cells were infected with MAP alone.

### 3.2. Time Course RNA Studies for IL-1β and IL-6

**Figure 2:**
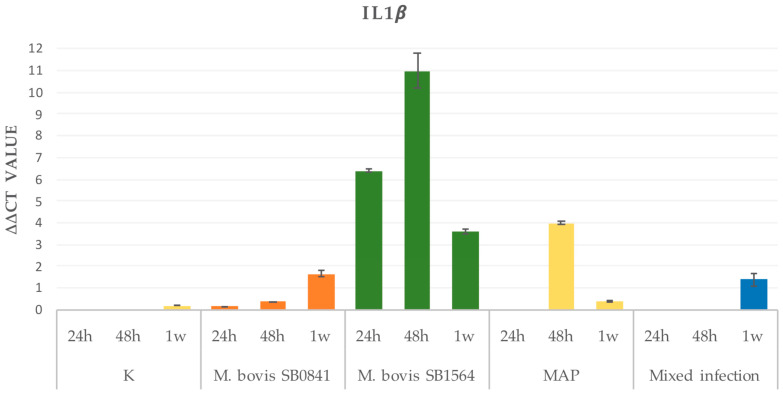
IL-1β gene expression on non-infected cells (K) and cells infected at maximum concentration of *M. bovis* SB0841, *M. bovis* SB1564, MAP and both bacteria (mixed infection) obtained by qRT-PCR 24 h, 48 h and one-week post-infection.

In order to define the time course of gene expression for IL-1β and IL-6, RNA analysis was performed at 24 h, 48 h and one week after infection with the highest bacterial concentration. Results are illustrated in Figure 2.

#### 3.2.1. IL-1β

Overall, uninfected cells revealed a progressive increase in RNA levels from 24 h to one-week post-infection. Interestingly, a similar increase rate was mirrored in the SB0481 and only after one week in the mixed infection. As for the *M. bovis* SB1564 infection, a strong gene expression was detected 24 h post-infection, reaching maximum levels at 48 h, and returning to lower levels one week after. A similar trend was evident for the MAP infection; however, no cytokine gene expression was evident 24 h post-infection. Subsequently, a peak was detected at 48 h, with return to basal levels 1 week post-infection (Figure 2).

#### 3.2.2. IL-6

**Figure 3:**
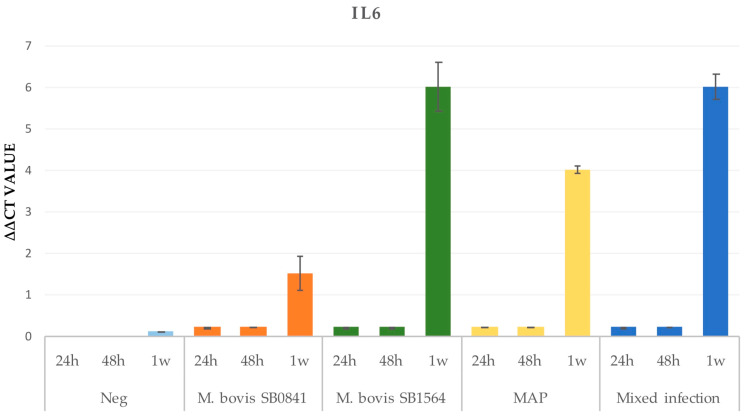
IL-6 gene expression on non infected cells (K) and cells infected at maximum concentration of *M. bovis* SB0841, *M. bovis* SB1564, MAP and both bacteria (mixed infection) obtained by qRT-PCR 24 h, 48 h and one-week post-infection.

The IL-6 gene expression appeared relevant only after one week post-infection for all the strains, with the highest levels detected for *M. bovis* SB1564, followed by the mixed infection, the MAP infection and *M. bovis* SB0841 infection. However, it is possible to observe a mild gene expression at 24 and 48 h post-infection. No expression was registered in the uninfected cells (Figure 3).

### 3.3. Plasma Cytokines Measurement

During the different time points (24 h, 48 h, and 7 days post-infection), a variation was detected for IL-6, IL-1β, considering the three different concentrations.

#### 3.3.1. IL-1β

**Figure 4:**
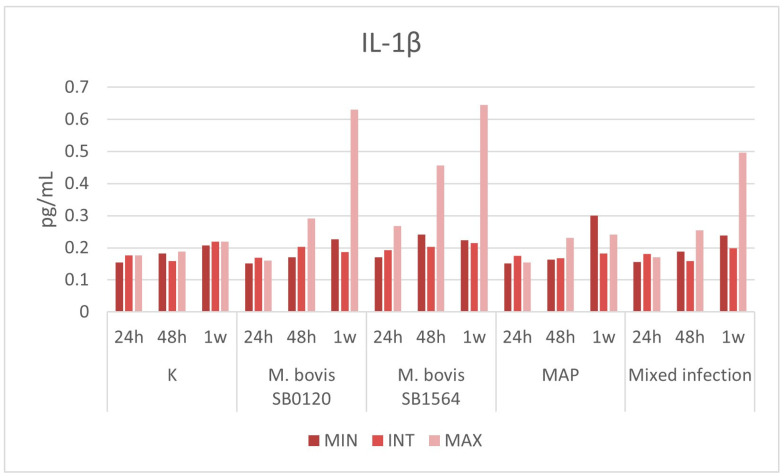
Plasma IL-1β levels (pg/mL) produced by macrophages infected at three different concentration of *M. bovis* SB0120, *M. bovis* SB1564, MAP and mixed infection 24 h, 48 h and one-week post-infection. Cytokine production on non-infected cells (K) is also displayed. MIN: minimum concentration; INT: intermediate concentration; MAX: maximum concentration.

Plasma cytokine levels remained stable over time in uninfected samples, not exceeding 0.22 pg/mL, which was the maximum value obtained after one week in the intermediate challenge. *M. bovis* SB1564 (0.268 pg/mL) and *M. bovis* SB0120 (0.203 pg/mL) had a slight increase in IL-1β production one day and two days post-infection, respectively (Figure 4). The levels of the cytokine increased with time, reaching the peaks of 0.645 pg/mL and 0.631 pg/mL one week post-infection at the maximum concentration of *M. bovis* SB1564 and *M. bovis* SB0120, respectively. MAP infection induced a slight increase in the IL-1β production after one week, with the production of 0.3 pg/mL at the minimum concentration. Lastly, mixed infection determined an increase after one week at the highest concentration, with the production of 0.497 pg/mL (Figure 4).

#### 3.3.2. IL-6

**Figure 5:**
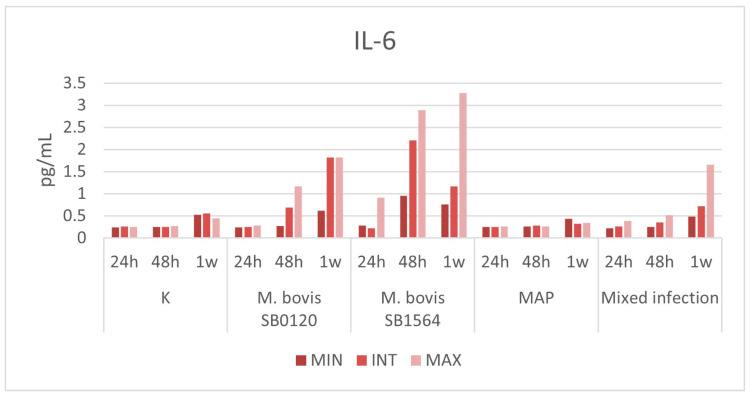
Plasma IL-6 levels (pg/mL) produced by macrophages infected at three different concentration of *M. bovis* SB0120, *M. bovis* SB1564, MAP and mixed infection 24 h, 48 h and one-week post-infection. Cytokine production on non-infected cells (K) is also displayed. MIN: minimum concentration; INT: intermediate concentration; MAX: maximum concentration.

The overall cytokine production in samples infected with *M. bovis* SB0120 and *M. bovis* SB1564 showed a progressive increase accordingly to bacterial concentration that starts 24 h post-infection and reaches a peak of 1.823 pg/mL and 3.273 pg/mL one-week post-infection at the maximum bacterial concentration, respectively (Figure 5). Production of IL-6 was higher in samples infected with *M. bovis* spoligotype SB1564 compared with spoligotype SB0120. The latter did not cause any increase in cytokine concentration until 48 h post-infection whilst spoligotype SB1564 was able to produce detectable levels of IL-6 (0.914 pg/mL) as soon as 24 h post-infection.

Mixed infection produced sensible levels of the cytokine at maximum concentration only 1 week post-challenge (1.655 pg/mL)-infection.

Interestingly, IL-6 production was detected during infection with MAP strain at any bacterial concentration and at any time point within one week, similarly to uninfected samples.

## 4. Discussion

Host-pathogen interaction due to mycobacterial infection elicits a complex response, with the involvement of a plethora of cytokines, in a delicate balance of stimulation and inhibition of the immune system. Our study aimed to evaluate the production of a panel of cytokines secreted by bovine macrophages in response to an in-vitro infection with MAP and *M. bovis* strains alone or in mixed infection.

### 4.1. Activation of the Immune Response in Relation to the Pathogenicity of Mycobacteria: The Modulating Effect

Marino et al., 2017 [33] observed an inhibition of the genes responsible for the activation of the immune response, therefore a greater survival capacity of MAP within the host cell after 2, 6, and 24 h post-infection of bovine macrophage lines. Similarly, Coussens et al. [34] showed a reduced activation of the immune system after in vitro MAP infection of peripheral blood mononuclear cells (PBMC), in particular in the clinical forms. Alfonseca-Silva et al., 2016 [35] found out that the production of a precise inflammatory pattern, both in terms of cytokines and macrophage receptors, is associated with a better control of *M. bovis* infection and bacterial growth. The authors observed that the degree of the inflammatory response is linked to the virulence of the strain in the *M. bovis* infection. In details, ELISA results revealed an intra-specific variability of IL-6 production between the two strains of *M. bovis* tested (SB0120 and SB1564), with a greater secretion observed in the *M. bovis* SB1564 infection. Therefore, the strain SB0120 seems to elicit weaker stimuli to the immune system and consequently the inflammatory production, while the less virulent strain (SB1564) is associated with a more effective immune response. These results could partly explain the reasons why SB1564 is a spoligotype less widespread and isolated than SB0120, towards which a greater pathogenicity is suspected given its ubiquitous character, isolation in different species and the ability to mutate rapidly [6]. Macrophage cultures infected with MAP did not register any increase in the aforementioned cytokine production despite evidence of gene expression, suggesting that MAP tends more easily to hide and inhibit an immune response (anti-inflammatory activity). Finally, the mixed infection showed intermediate values between those obtained by the two bacteria separately, suggesting the existence of a modulating activity by one or both towards the activation of the monocyte-macrophage cell line at least in the initial stages of the infectious process.

### 4.2. IL-6

IL-6 is a cytokine with pro and anti-inflammatory functions produced by fibroblasts and by cells of the monocyte-macrophage lineage. It plays multiple roles in different phases of the immune response (innate, acquired). It stimulates the synthesis of acute phase proteins and neutrophils and recruits macrophages to the site of infection. This cytokine is usually produced following the interaction of macrophage receptors PAMPS (pathogen-associated molecular patterns) with some bacterial microorganisms [36]. IL-6 acts inhibiting lymphocyte apoptosis and the differentiation of T regs whilst promoting the production and differentiation of Th17 and B lymphocytes. It is also implicated in non-inflammatory processes, such as neoplasms and muscular activity (myokine) [37,38,39,40,41]. The early and progressive increase in the IL-6 production was detected by qRT-PCR and confirmed by the ELISA test. The early involvement of this cytokine in the mycobacterial infections has been already observed by Singh and Goyal [25] who indicated it as one of the most powerful biomarkers for detecting *M. tuberculosis* infection. Interestingly, in our study, spoligotype SB1564 induced the highest levels of IL-6 production. Conversely, the cytokine production induced by SB0841 was overall lower. IL-6 and TNFα have been associated with the formation of granuloma and chronic wasting syndrome in paratuberculosis but their role is not yet clearly defined [42]. However, a study conducted on a modern culture system aimed at mimicking the evolution of the MAP infection has highlighted how in the initial phases MAP induces a mild or absent immune response to which it is associated a particular lipid phenotype. In the most advanced stages of the disease, when the mycobacterium has had the opportunity to settle in the target cells and multiply, a change in the structural composition follows, presumably linked to metabolic needs, which further stimulate an activation of the cells of the immune system and the development of a well-defined cytokine pattern [43].

### 4.3. IL-1β

IL-1β is a powerful pro-inflammatory cytokine essential for the host response to microbial infections. It is secreted by various cell populations but above all by monocyte-macrophages. Its activation mechanism is unclear since it does not follow the conventional cascade path common to all cytokines but appears to be released continuously. This could explain the tissue damage that the latter produces both in the acute and chronic phases of the infection [44]. This cytokine activates macrophages and neutrophils and induces an adaptive response where Th1 and Th17 predominate [45]. A study conducted by Bourigault et al. [46] on the role of IL-1β and TNFα with *M. tuberculosis* and *M. bovis* strains highlighted how both cytokines are essential for the control of the infection. However, while TNFα is effective in the early stages of both strains, IL-1β does not play a leading role in acute *M. bovis* infection.

The results obtained for this cytokine by the ELISA test showed a limited production starting from 48 h following infection for both *M. bovis* strains with all the three different concentrations that appeared relevant only one week post-infection at the maximum bacterial concentration. In the case of MAP, a slight increase was observed after one week and with the minimum bacterial concentration. These results may suggest that, while for *M. bovis* the concentration of mycobacteria is essential to exponentially stimulate the production of IL-1β and the induction of the inflammatory response, MAP infection induces a macrophage activation not strictly related to the number of bacteria, as it appears to occur with the maximum microbial dilution. The qRT-PCR results showed a peak in IL-1β transcript for *M. bovis* SB1564 strain, as observed with the ELISA test, and MAP while it appeared reduced with *M. bovis* SB0841 and absent in the mixed infection with eventually a peak at one-week post-infection.

### 4.4. IL-2

IL-2 is primarily produced by activated CD4+ T cells, CD8+ T cells and dendritic cells and it is implicated in the proliferation, differentiation and survival of CD4+ and CD8+ contributing to the activation of an antigen-specific immune response [47]. This cytokine was differently expressed in all the infections without following a specific pattern. Its significance needs to be further investigated.

### 4.5. TNFα

TNFα is a cytokine produced mainly by macrophages but also by other cell types, such as lymphocytes, endothelial cells, and fibroblasts. Besides being involved in the inflammatory process, it is involved in different biological phenomena, such as cell apoptosis, differentiation, and carcinogenesis. In the course of mycobacterial infections TNFα induces the expression of chemokines, such as IL-8, MCP-1 and RANTES that send signals for the migration of immune system cells to the sites of infection. It acts also in synergy with IFN-γ to enhance the antimycobacterial activity of macrophages. Finally, it guarantees long-term protection against mycobacterium [48].

qRT-PCR tests of this cytokine have shown one week after infection and at maximum concentration, a basal gene expression in all three types of infection, albeit at different levels. The highest levels of inhibition were achieved with the *M. bovis* SB1564 strain, followed by the *M. bovis* SB0841 strain, that is another extremely diffuse strain that has been isolated across different species in Sicily [2], MAP and finally mixed infection. The basal gene expression maintenance induced by mycobacteria against this cytokine could be a survival mechanism implemented by the pathogen within the host cell [49].

### 4.6. IL-10

Interleukin 10 is involved in the maintenance and homeostasis of tissues. In this regard, it promotes the innate immune response to limit the damage induced by any viral and/or bacterial infections and also acts as a powerful anti-inflammatory to limit the destructive activity induced by immune processes [50]. In mycobacterial infections, IL-10 promotes the survival of mycobacteria through a series of actions that provide the inhibition of the maturation of phagosome, the reduction in nitric oxide production, the blocking of the IFN-γ signal. It also antagonizes the Th1-type lymphocyte response by inhibiting the presentation of the antigen and the production of IL-12. It is generally increased during tuberculosis infection [28]. Contrary to what is reported in the literature, the analysis conducted by qRT-PCR on bovine macrophage cells revealed an inhibition of the gene expression of the aforementioned cytokine (in all three types of infection at one week and at the maximum concentration), which was greater with the *M. bovis* strains followed by the mixed infection and MAP. This inhibition corresponds to the intense pro-inflammatory response represented by IL-6 and in part by IL-1β and IL-12 (transcript).

### 4.7. IL-12

It is a cytokine produced mainly by cells of the macrophage system. It acts in different phases of the immune response. In the initial stages of infection by inducing the production of IFN-γ by T and NK lymphocytes which contribute to macrophage activation and phagocytosis, it supports the differentiation of lymphocytes into CD4+ and constitutes a functional bridge between innate and acquired immunity [51]. The results obtained in qRT-PCR indicate an increased gene expression in both *M. bovis* strains (with a clear prevalence for SB1564) and in mixed infection, vice versa it appears inhibited with MAP. Further studies are required to evaluate the correlation between cytokine production and gene expression and its potential as differential diagnostic marker.

### 4.8. IFN-γ

IFN-γ plays a leading role in the immune response (mostly immunomodulatory activity) to various viral and bacterial pathogens. The primary producers are T lymphocytes and Natural Killers; however, this cytokine is also produced by macrophages [52]. Its presence is associated with an effective resolution of the infectious process by the immune system [53]. The qRT-PCR detected high levels of IFN-γ transcript one week after infection in macrophages infected with MAP and in the mixed infection. There is no clear explanation for this, and further tests are required.

## 5. Conclusions

- The variability of the macrophage response not only linked to different bacterial species *M. bovis*/*M. paratuberculosis* (interspecific) but also intraspecific (two different spoligotypes) which could also be correlated to factors such as strain pathogenicity and virulence. In particular, the comparison in gene expression and cytokines measurements among spoligotypes, revealed a stronger immune stimulation given by SB1564 when compared with both SB0841 and SB0120, respectively. The differences found corroborated the hypothesis that immune response to SB1564 might be more effective, thus partly explaining the reason why this spoligotype is less widespread compared with others.
- The variability of the immune response is associated with the bacterial concentration especially for *Mycobacterium bovis.* Exposure to high levels of bacterial concentration may enhance the immune response and facilitate the identification of the infection through the diagnostic tests whereas low levels of infection may not be detected by the official tests and persist within the infected area for a longer time allowing the spreading of the mycobacteria. Some *M. bovis* strains appear to produce a reduced/delayed inflammatory reaction similar to what observed with MAP potentially showing the same ability to “camouflage” within the host cell.
- The identification of IL-6 as an early marker of bovine tuberculosis in the bovine species. The different production noticed in the MAP infection may suggest that this bacterium stimulates the host cell to induce an inflammatory pattern only after a longer period of incubation so even one week was not sufficient to induce a clear cytokine production. This hypothesis is supported by the detection of the transcript by qRT-PCR analysis. Another interesting point is related to the pathogenic aspect. Its massive production, as it can happen in chronic inflammatory processes, induces an imbalance and an unfavourable outcome (e.g., autoimmune diseases such as multiple sclerosis and Chron’s disease). This suggests how mycobacteria can influence and determine the onset of these diseases by stimulating this type of response. It also stimulates the production of B-lymphocytes notoriously ineffective against the tuberculous infections.
- The possible phenomenon of inhibition or modulatory effect observed and induced by the copresence of the two microbial species (MAP and *M. bovis*) may lead to important implications in the application of the eradication plans. It could be considered as evidence supporting the hypothesis that the coinfection in the same animal interferes with the response to the official diagnostic tests and therefore with the outcome of the test, contributing to the maintenance of *M. bovis* infection in the farms.
- The presence of a primary transcript attributable to IL-2 in macrophages in both infections.
- The lack of correlation between gene expression and cytokine production (ELISA test). It can be partly explained by the macrophage cell and its post-transcriptional modifications. In fact, there is no direct correlation between the RNA produced and protein synthesis. Possible explanations are related to species characteristics, the complex mycobacterium/cell interaction that influences the production and/or inhibition of some cytokines and other aspects such as survival mechanisms and/or proliferation within the host cell, or finally to possible regulatory and inhibitory feedback mechanisms [54].
- The inhibitory action on the TNFα transcript induced by both mycobacteria regardless of the strain can potentially be interpreted as a survival mechanism.
- The inhibition of the gene expression of IL-10 during the early stages of the disease whilst notoriously increased in the most advanced stages of tuberculous infection may be linked to the need of the mycobacteria to recruit more macrophages as host cells where they can hide and multiply.
- Inhibition of IL-12 production as a possible further attempt made by MAP to delay the activation of the immune response.

In conclusion, the present work provides some insights that not only help to clarify some aspects of the unclear pathogenic mechanisms of the two infections, but also provide some diagnostic implications.

## Figures and Tables

**Table 1:** Gene-specific primers used to amplify fragments of Bos taurus genes for cytokine expression.

Gene Name	Accession Number	Forward Primer 5′–3′	Reverse Primer 5′–3′
IL-1β	NM_174093.1	CTAGCCCATGTGTGCTGAAG	CCACTTCTCGGTTCATTTCC
IL-2	NM_180997.2	ACTCCTGCCACAATGTACAAG	TGTTCCCCGTAGAGCTTGAA
IL-6	NM_173923.2	TAACACCATCAAGGACCTGT	TGCCCAGGAACTACCACAAT
IL-10	M_174088.1	ACTCTGTTGCCTGGTCTTCC	GACAGGGTGCTCGCATCT
IL12p40	EU276076.1	TTGCTCTCAGCAGAGAAGGTC	CTGCCCTCCTGACACTCC
IFN γ	NM_174086.1	GGCATGTCAGACAGCACTTG	TGAAGCGCCAGGTATAAGGT
TNFα	AF348421.1	GTGTGAAGCTGGAAGACAACC	CCCTGAAGAGGACCTGTGAG
GAPDH	NM_001034034.2	AGATGGTGAAGGTCGGAGTG	GACGATGTCCACTTTGCCAG

## Data Availability

All the research data are available in the submitted manuscript file.
